# Exploring the Experiences of Individuals Diagnosed with Metastatic Non-Small-Cell Lung Cancer: A Qualitative Study

**DOI:** 10.3390/curroncol32100570

**Published:** 2025-10-15

**Authors:** Sarah Scruton, Caroline Hovey, Cynthia Kendell, Robin Urquhart

**Affiliations:** 1 Cancer Outcomes Research Program, Nova Scotia Health, Halifax, NS B3H 1V7, Canada; sarah.scruton@nshealth.ca; 2 Department of Community Health and Epidemiology, Dalhousie University, Halifax, NS B3H 1V7, Canada; cr344200@dal.ca; 3 Department of Medicine, Dalhousie University, Halifax, NS B3H 2Y9, Canada; cynthia.kendell@nshealth.ca

**Keywords:** lung cancer, metastatic, qualitative research, supportive care, survivorship

## Abstract

**Simple Summary:**

Improvements in cancer treatments have led people with metastatic non-small-cell lung cancer (mNSCLC) to live much longer than before. However, most existing support services are designed for patients diagnosed at an earlier stage who were treated with the goal of curing their cancer, and do not fully meet the needs of those living long term with metastatic cancer. In this study, eight Canadians with mNSCLC shared their experiences. While generally satisfied with their medical care, they experienced little support for side effects, emotional challenges, and practical challenges (i.e., financial burdens). Existing supportive care programs (i.e., exercise programs) were appreciated but often provided as short-term services, were difficult to access, and required patients to find them on their own. This study shows the need for supports tailored to those with mNSCLC, better healthcare coordination, and expanded resources. These findings can guide future research and the development or adaptation of resources to help address these challenges.

**Abstract:**

Advancements in targeted therapies and immunotherapies have improved survival for individuals with metastatic non-small-cell lung cancer (mNSCLC), creating a growing population of Canadians living long-term with the disease. These individuals face ongoing physical, emotional, and practical challenges, yet existing supportive care services are often designed for patients receiving curative intent treatment and may not adequately address the challenges of those undergoing continuous treatment. To explore these experiences and inform the development of supports tailored to their needs, eight participants with mNSCLC completed one-on-one virtual interviews. They described limited support for managing side effects and psychosocial concerns despite general satisfaction with oncology care. Fatigue and cognitive challenges impacted daily functioning, and emotional challenges (e.g., fear of progression, stigma, and difficulty finding meaning) impacted quality of life. Financial burden, including unexpected costs and loss of income, further affected their well-being. Existing supports, such as exercise programs, were viewed positively but were often difficult to access, were offered only short-term, and required patients to find them independently. Recommendations included improved coordination and communication across the healthcare system, alongside tailored interventions such as navigation services, resource directories, health promotion supports, and expanded peer support. Overall, people living long term with mNSCLC face distinct challenges and unmet supportive care needs, highlighting the importance of integrating supportive services into routine oncology care.

## 1. Introduction

Approximately 32,000 Canadians are diagnosed with lung cancer every year [1], with non-small-cell lung cancer (NSCLC) accounting for 80–85% of cases [2]. Until very recently, the prognosis for those diagnosed with metastatic NSCLC (mNSCLC) was very low, with worldwide 5-year survival being just 6% in 2016 [3]. Over the past decade, however, the rapid advancement of precision oncology has led to treatments that are personalized based on a patient’s specific molecular profile [4]. These novel targeted therapies and immunotherapies have led to unprecedented improvements in survival [4,5]. For example, targeted therapies such as Tyrosine Kinase Inhibitors (TKIs) have improved outcomes 10-fold in the treatment of Anaplastic Lymphoma Kinase (ALK+) mNSCLC, with 5-year survival increasing to 62.5%. Such advances have led to a new population of Canadians who are now living long-term with mNSCLC [6].

Despite these improvements in survival, individuals living long-term with cancer often face physical, practical, and psychosocial challenges resulting from their cancer and ongoing treatment, without a defined endpoint [7,8]. These challenges likely impact their daily functioning and may greatly impact their quality of life. For those with mNSCLC, we do not currently have an understanding of the specific challenges faced by this population and how they may differ from others with cancer (e.g., people with early-stage cancers and those treated with curative intent). However, a small number of studies have found that this population experiences a higher and more persistent symptom burden compared to those receiving curative-intent treatment [9,10,11]. This has not yet translated into the development of supportive care interventions targeting individuals living with mNSCLC who will be on treatment indefinitely. Existing resources and supports are most often designed for those who are on treatment short term who will be re-integrating in the context of no evidence of disease [12]. These interventions tend to be provided on a short-term basis and focus on goals such as physical rehabilitation or returning to work [12,13]. As such, individuals living with mNSCLC are faced with trying to manage the long-term effects of their cancer and ongoing treatment on their own or accessing supports that are only offered temporarily and of limited relevance.

To improve the quality of life of the growing population of individuals living long-term with mNSCLC, we must begin to identify the unique challenges of this population and develop supportive care interventions that are specifically geared to meet these challenges. To date, survivorship research has largely centered on individuals with early-stage disease or those at the end of life. Lai-Kwon et al. emphasized the need to broaden this focus and expand on the 2006 and 2013 Institute of Medicine report recommendations to include people living with metastatic cancer [14,15,16]. Their recommendations highlight the importance of strengthening communication and care coordination, redefining survivorship models, and ensuring that survivorship research meaningfully incorporates the voices and experiences of metastatic cancer patients [14]. As such, this study sought to explore the lived experience of those receiving targeted therapies or immunotherapies for mNSCLC, and to identify the components of potential interventions to improve care and support for this population.

## 2. Materials and Methods

### 2.1. Participants and Recruitment

We aimed to conduct 8–10 one-on-one interviews with individuals from across Canada who were diagnosed with mNSCLC, continuing recruitment until saturation was achieved. Participants were eligible if they were above the age of 18, English-speaking, and receiving long-term targeted therapies or immunotherapies. Participants were recruited through posters shared across social media, including mNSCLC support groups and organizations who work closely with lung cancer patients. The posters included the contact information for one author (SS) in charge of recruitment, who interested individuals contacted via email or phone number. Once eligibility was confirmed, the participants consented to and were enrolled in the study.

### 2.2. Data Collection

Data collection involved 1 h semi-structured interviews using video-conferencing software (Zoom for Healthcare, version 6.9.5). The interviews were conducted by the Research Coordinator who is Master’s trained and experienced in qualitative methods [SS]. The interview guide was developed by the research team, based on the study objectives and overarching guidance from Patton [17] and Rubin and Rubin [18] on structuring interviews and questions (Supplementary File S1). The interview guide was not piloted. However, the guide was used in an iterative manner, meaning it was adapted as/when needed based on the initial responses of the study participants. The research team did not have prior personal or professional relationships with the study participants. The open-ended questions were designed to capture: (1) participants’ experiences living with mNSCLC; (2) the specific physical, emotional, and practical challenges of this population and how these challenges were addressed (or not); and (3) participants’ views on the interventions (or intervention components) required to best address these challenges. All interviews were audio-recorded and transcribed verbatim. No repeat interviews were conducted.

### 2.3. Data Analysis

The analysis was guided by qualitative description as described by Sandelowski [19,20]. Using this approach, the contents of the data are summarized and described with minimal interpretation. This involves coding the data while recording insights and reflections, sorting the data to identify unique and similar concepts, extracting and refining themes, and comparing findings to existing knowledge and literature [19,20]. A coding framework was developed by SS, in collaboration with two additional co-authors [CH, RU], to guide the organization of the data. Coding was both deductive and inductive, as codes were added or consolidated based on the findings. Prior to analysis, identifying information was removed from the transcripts. The first two transcripts were analyzed by both SS and CH to ensure consistency, refine the coding framework as needed, and resolve any discrepancies or disagreements. The remaining transcripts were then divided and coded independently by SS and CH, under the supervision of the primary investigator [RU] who has extensive qualitative research experience. The coded data were then refined and examined by SS and CH, supervised by RU, to identify recurring and distinct themes, explore commonalities and differences between participants, and capture relevant quotes to illustrate the findings. All researchers, including the interviewer [SS], are women with experience in cancer health services research. NVivo software (version 15) was used to facilitate coding and data management.

## 3. Results

Eight individuals diagnosed with mNSCLC (5 women, 3 men) from across Canada took part in interviews. They ranged in age from 40 to 77 years, with time since diagnosis spanning 1 to 11 years. Most (75%) had a Bachelor or Master’s level of education. All but two participants lived in urban or suburban areas, and the majority lived within a 30 min to 1 h drive to their cancer specialist. Detailed demographic characteristics are displayed in Table 1.

During the interviews, participants discussed their healthcare experiences and the challenges they faced following their diagnosis. They described the supports they found helpful, as well as barriers and facilitators to accessing those supports. They also highlighted unmet needs and suggested potential interventions to address these gaps in their care.

### 3.1. Current Healthcare Experiences

In general, participants felt confident in their treatment and the care they were receiving from their oncologist. They described positive relationships with their healthcare providers (HCPs), and those with family doctors appreciated their added support for non-treatment related issues. However, several participants noted that appointments tended to focus on discussing their cancer (i.e., scan results, treatment plans), with limited discussion of or supports for psychosocial needs or side effects. This was often attributed to a lack of time during appointments, or HCPs’ unfamiliarity with their specific cancer subtype (most often ALK+).

*“I think the main one, we talked about it almost as a thread through the whole works here, was the lack of oncology doctors to have enough time. And I’m not going to criticize any of them because they’re all wonderful across our nation and across the world. But having enough time to really sit down and talk to us in more of…not just medical all the time, but just like, How are you doing emotionally?”*

[P7]

### 3.2. On-Going Challenges

#### 3.2.1. Physical Challenges

Participants reported a range of physical challenges including pain, muscle loss, fatigue, gastrointestinal issues, reduced lung capacity, and sleep disorders. Fatigue and muscle pain/weakness were commonly described as ongoing challenges, as they continued to impact the participants’ ability to work, complete tasks without needing rest, or exercise. Similarly, ongoing cognitive issues such as brain fog, memory problems, and difficulty with speech also interfered with their ability to complete day-to-day activities.

*“Initially I couldn’t work because of physical pain that I had. And then I couldn’t work because of the fatigue and my mental capacity just wasn’t there as… I was working as a [career], which is very fast paced. And it just… My head wasn’t in the game anymore.”*

[P2]

#### 3.2.2. Emotional Challenges

Three key emotional concerns were discussed by the participants: fear and anxiety, stigma, and finding meaning. Fear of progression, described as “scanxiety” or living on borrowed time, was a source of distress. They feared what would happen when their current treatment stopped working and whether they could access further therapy, especially given limited provincially funded drug coverage and the financial strain of being unable to work.

*“I think the things that are concerning to me are the ones we already talked about earlier. And it’s particularly this access to second line medication and ulterior lines of medication. Because until 2021, I think it was, alectinib was accessible in Canada. But it was accessible because it was the pharmaceutical company paying for it. And, yeah, obviously they got fed up of paying for everything themselves, and the government didn’t kick in. So that wound down. And so today, everyone is an orphan. And I know some people who buy it privately. And the cost… I know one guy, he buys it, and the pharmaceutical company pays half, and he pays half. But it’s nevertheless almost $4000 a month. But not everybody has those means.”*

[P4]

*“But, you know, if you want to know the thing that worries me, is what happens… I can’t imagine the… I don’t know if agony is the right word. But just like… So, if the Lorlatinib stopped working for me, and there’s something else out there that’s working for people, but I can’t get it, right. I just can’t imagine that.”*

[P3]

Stigma was also widely reported; nearly all participants had never smoked, yet they felt judged due to the misconception that smoking is always the cause of lung cancer.

*I really do think there was a stigma there that, you know, why should we put money in there, they’re causing their own problems? Well, I never smoked, you know. So… No radon. We did check our house for radon. So, it’s not that. So, who knows? I don’t know*.

[P6]

They also felt misunderstood by others who saw cancer as a binary outcome resulting in cure or death, with no understanding of living long-term on treatment.

*“Like people see cancer as binary. You either die or you’re cured. They don’t understand that a large percentage of us are sort of in that in between, right? Like even when people are cured, now we’re finding that there’s always, you know, a lot of recurrence. Like it’s not always cured, cured. Like it comes back. So how to talk to people, and manage their expectations when it comes to like, I have cancer. “[Gasp]. Are you going to die? Can I talk to you? Can I come visit you? It’s like, No, I’m not…”*

[P1]

Leaving work or giving up their normal activities made it difficult to find meaning or a sense of normalcy, especially for those who felt mentally capable of continuing but were physically unable. Some turned to volunteer or advocacy work, but as one participant described, this was not always as fulfilling as their careers and posed a risk of threatening their disability benefits.

*“I was told that I can’t volunteer for so many hours per week because then it might look like I’m well enough to go back to work. But honestly, I have a job that was so stressful and required so much of my brain time. That just because I volunteer 15 h at a food bank, doesn’t mean I can do 15 h of my job anymore. Like it’s just not the same. Like doing a repetitive task at a volunteer job is not the same as like… So, like how to find meaning in your life? So, what’s next? How do you find meaning in your life if you’re not well enough to go back to work?”*

[P1]

Finally, fear of burdening loved ones sometimes led to isolation, especially when they felt they could not relate to others in their personal lives.

#### 3.2.3. Practical Challenges

Most participants discussed leaving the workforce due to the long-term effects of their cancer treatment. While two had already retired, the remaining participants experienced work transitions. Knowing they would likely remain on treatment indefinitely, those who had left work relied on long-term disability or personal savings which required significant lifestyle adjustments.

*“My private insurance lasts for another seven years. So, if I’m lucky enough to live another seven years, which I hope I am, what are we going to do then? Because, you know, we have some savings, but we needed to tap into that for our kids’ education and stuff. Because, you know, that extra money that I had hoped to be making that I referred to earlier, like that would have been used to help do that. So, we’ve had to tap into savings to do some of those things. And so… And we’re not in a situation where we can add to our savings. So, what am I going to… What are we going to do when I’m 65 and that pension runs out? I’ll probably have to go back to work.”*

[P3]

*“And there’s the financial impact of being in the situation. You know, from one end of the spectrum to the other on this. We had advisors telling us like, well, listen, you know, you get a disability, you know, you probably, you’re either going to go on disability or die. So, you try to deal with both possibilities. But, you know, what about needing to go on disability but not dying and living longer, and just how to live? Having to figure that out financially which is a little bit of a challenge.”*

[P3]

The financial burden was exacerbated by the unexpected cancer-related costs, including both time and money spent traveling for appointments. For example, those who had temporarily or permanently lost the ability to drive had to rely on friends and family or paid transportation. One participant expressed fear that if they did drive, their cancer diagnosis could be used against them by insurance companies in the event of an accident.

### 3.3. Facilitators and Barriers to Challenges Being Addressed

Participants described several barriers and facilitators that influenced whether their challenges were addressed. Importantly, the same factor might sometimes act as both a facilitator and a barrier depending on how it was experienced. For example, HCPs helped patients overcome challenges when they offered open communication and appropriate referrals. At the same time, a sole focus on the medical aspects of managing the cancer and poor coordination between HCPs were significant barriers.

#### 3.3.1. Facilitators to Challenges Being Addressed

Participants identified supports and facilitators that helped to address their challenges both within and outside of the healthcare system. Healthcare supports included palliative care, physiotherapy, social workers, dietitians, therapists, and home care. Participants felt supported when physicians were open to discussing needs, learning about their cancer sub-types, and making appropriate referrals.

*“You know what, my palliative care doctor is probably the best resource there is. Like she’s amazing. So if I just send them a note, they’ll probably be able to help me out with referrals and stuff like that, and trying to find the things that I need.”*

[P1]

*“I was stable. So, then I received all of my care in that manner from my family doctor. Who of course had never heard of my particular kind of cancer or the drug that I was on. But between the two of us, we devised a pretty good system of how I would move forward with my day-by-day ailments that were caused from the drug.”*

[P2]

Peer support, especially through Facebook groups, was vital for providing emotional support, information sharing, opportunities to give back, and reducing isolation. Friends and family provided both emotional and practical support, such as helping to manage daily tasks.

*“My family is very supportive, and they were very helpful. You know, they brought food up. And I had more soup than I could deal with, you know. So, my husband could just focus on everything that he had to do with me. So, they’re very helpful”*

[P6]

Financial support through private insurance, savings, income from a partner, or ongoing employment helped reduce stress and improve access to care. Exercise programs were often highlighted for helping manage symptoms and rebuild strength. Finally, personal traits such as optimism, self-advocacy, and being actively engaged in one’s care were seen as important to coping and addressing their challenges.

#### 3.3.2. Barriers to Challenges Being Addressed

The most commonly reported barrier was a lack of meaningful support from HCPs. Participants felt their HCPs were focused solely on treating their cancer, with little support provided for holistic needs.

*“Well, in the beginning, I had physical symptoms from the cancer. And then after I started treatment, things were healing and I didn’t have the physical symptoms. But then I had a lot of side effects from the drugs. And my oncology team did not address any of it. Because my perception is they only cared about what was going on with the scans and how the body was looking, you know”.*

[P2]

*“You know, once you can go past, you know, one year on the calendar, I think there’s a psychological benefit to that. And, yeah, I would have liked somebody to help me at that time. But as it turned out, there was nobody. So, you know, I just quietly muddled through that, and perhaps came out stronger at the end, I don’t know.”*

[P4]

Referrals to psychosocial support, dieticians, exercise programs, or similar supportive care services were rarely offered by cancer specialists, yet participants felt they would benefit from these services. Participants also discussed receiving little information about their cancer or treatment from their HCPs and instead turning to their peers. Lastly, they noted poor coordination between HCPs, especially when it came to oncologists coordinating with non-traditional professionals like dieticians or naturopaths. Some available supports were only offered short term or were poorly suited to those living long term with advanced cancer.

*“So [social worker] mentioned the [University] has a program for people that once they complete their treatment, to help get them back up… To get back into normal activity, is the idea behind it. But again, I think it’s because it’s for people that are done treatment and on the other side of things. So, I don’t think that I quite qualify for that yet because I’ll be in ongoing treatment.”*

[P8]

As a result, the participants often described having to search for supports themselves without knowing what was out there or what to trust, which made them feel overwhelmed as they did not feel this should be their responsibility.

### 3.4. Proposed Healthcare Supports and Intervention Development

Participants identified a range of supports that could better address their needs and reduce the challenges they encounter. Some require adjustments to the healthcare system and may include policy-level change, whereas others may require the development of new interventions or the adaptation of existing ones.

#### 3.4.1. Healthcare- and Policy-Directed Changes

Participants discussed the need to improve access to timely diagnosis and treatment as delays in biomarker testing can lead to delays in receiving appropriate targeted therapies, which have the potential to greatly improve survival. Funding barriers for targeted treatments were also a major concern, and participants stressed the need for advocacy work to hasten regulatory approvals and provincial coverage of therapies.

The need for improved communication within the healthcare system was discussed regarding better communication between HCPs, and clearer communication between HCPs and patients about their cancer and its treatment. Participants wanted access to holistic care and automatic referrals to additional supports such as psychologists, massage therapists, social workers, or naturopaths. They felt their care would be improved using a collaborative, integrative approach where HCPs communicate and work together to treat the whole patient rather than only treating their cancer.

*“Well, that’s my fantasy world—that I would like to see different practitioners working together, non-traditional as well as the traditional. That there was some way to bring them together in a collaborative setting. Because to me, they complement each other. And I think that, you know, I would have appreciated not having to kind of tiptoe around some people and make sure I asked nicely. You know, that it should just be automatic. Like these people have been here forever—osteopaths, massage therapists. And like I was told I couldn’t do it. And honestly, the treatments are so gentle that I could have been doing it all along.”*

[P6]

#### 3.4.2. Access and Information

A suggestion commonly mentioned by participants was that it should not be the patients’ responsibility to seek support for their challenges. Participants described two discrete interventions they felt would address this need. Firstly, they recommended access to a dedicated patient navigator (such as a nurse) or social worker who could point them in the direction of available supports and services.

*“I think it’s called a nurse navigator or something. Apparently in the States they have them where somebody is with you and can kind of guide you through all of these things. Say, you should see this, you should go here. That might be helpful. And it maybe doesn’t even have to be a nurse. But if there’s somebody that knows all of the things, can check all the boxes, then, you know, you’re covering all your bases”.*

[P6]

Secondly, they suggested the development of a centralized, easily accessible, and regularly updated “one-stop-shop” of information on available supports and mNSCLC-related information. In the form of a website or app, this could be provided to them at the time of diagnosis and used when needed to provide just-in-time information. Some participants also recognized the usefulness of a physical copy of information (such as a binder) for those who are not comfortable with technology.

*“I would love to have some kind of one-stop shop. Like, you know, start here, and then if you want more information, like here’s all the different things you could look for, and then you could like branch out there. But it would be great to kind of have one place where it’s like you start here now. Versus like us trying to find, you know, different things in different places”.*

[P1]

*“Well, I mean day one when I was diagnosed, I feel I should have been given at least a pamphlet describing what this cancer was, how it worked, and a direction on how to get a support group if… I know not everybody’s interested in a support group, but we need to be made aware of what the options are. A therapist immediately to discuss with the family, or not, I feel is critical. Eventually it would become exercise programs and things like that”.*

[P2]

#### 3.4.3. Overall Wellness and Maintaining Function

Participants also emphasized the importance of interventions focused on health promotion and overall well-being. They wanted programs highlighting the benefits of exercise, healthy living, and proactive symptom management or prevention. They suggested how these interventions could be tailored for those with mNSCLC with a focus on improving lung capacity, managing weight changes, maintaining mobility, and increasing strength while remaining safely within their limits. This could also include nutrition guidance to support function and promote health. Other suggestions for supports included financial planning resources, end-of-life care and information, assistance with travel, and further opportunities for peer support tailored to those on treatment long-term.

*“Yeah, because physical health is one thing that… Because I don’t… I’m really trying to maintain and not decline. It’s not even a goal of improving at this point, right? Like I just want to like not decline. So, I certainly want to incorporate physical activity. And I have been in… we have some equipment in the basement. But I don’t know how much to push myself. So, yeah, so it would be nice to have a resource. Like somebody that would work with people in treatment. Because, like you said, I’m going to have to learn to live with, right, and develop my new normal with living with cancer, kind of thing.”*

[P8]

Finally, participants described key considerations to keep in mind when designing these interventions. Timing was important, as patients may be overwhelmed at the time of diagnosis and forget about the supports offered to them. Therefore, supports should be offered at different time points and should be on-going. In addition, interventions should account for differences in age, comfort with technology, family responsibilities, and travel ability.

## 4. Discussion

This study describes the ongoing challenges and needs experienced by those living long-term with mNSCLC. Given the small sample size (*n* = 8), of relatively highly educated individuals, the results of this study may be of limited generalizability to the broader population of mNSCLC patients in Canada. Nonetheless, given the paucity of literature focused on the growing population of individuals who are living long-term with mNSCLC, this study provides important insights on a growing patient population and their unique healthcare needs.

Overall, despite their cancer being well-managed by their treatment and healthcare team, participants felt that their holistic needs (i.e., physical, emotional, and practical) were not being addressed and continued to disrupt their daily lives. Fatigue and brain fog prevented them from completing day-to-day tasks, whereas anxiety, stigma, and difficulty finding meaning caused them emotional distress. Practical challenges were discussed in relation to leaving work and unexpected financial instability. Participants faced difficulties accessing supportive care, worsened by a lack of referrals and guidance and few interventions geared towards those on long-term treatment. They felt that better communication and collaboration between oncologists and supportive care providers (i.e., psychologists, nutritionists) is essential. Participants also suggested the development and adaptation of other interventions that could help to address their challenges, including navigation support, resource directories/hubs, health promotion and exercise programs, and further opportunities for peer support. Such interventions have shown success for other cancer types and stages of disease, resulting in improved quality of life, well-being, social functioning and confidence, and decreased isolation [21,22,23].

Many of these challenges have been reported elsewhere amongst several cancer types and stages. A Canada-wide survey amongst adult cancer survivors found high levels of unmet needs, with 81% of respondents reporting unmet physical concerns, 78% reporting unmet emotional concerns, and 74% reporting unmet practical concerns [24]. Consistent with our findings, the most common needs in each of these categories included fatigue and brain fog, anxiety, and getting to and from appointments, respectively. Previous studies have also reported similar healthcare system barriers to addressing challenges. For example, a worldwide study including 37 countries found that patients rarely described psychosocial care as a part of their cancer care [25]. Likewise, a review by Dilworth et al. found that aside from patients reporting a personal choice not to access support, the second most common barrier to accessing services was a lack of information from their HCPs [26]. Together with the findings of the current study, this points to a clear communication gap where patients are uninformed of existing services that may address their challenges.

While there are certainly many similarities in the experiences of individuals with mNSCLC and other populations of cancer patients and survivors in terms of the challenges and needs being faced, individuals with mNSCLC face distinct and persistent difficulties that differ from those treated with curative intent. This means that in addition to the communication barriers identified for other cancer types, there is also a general lack of supports personalized to their needs. Unlike post-treatment survivors, they remain on continuous treatment without a clear end point, meaning there is no “post-treatment” transition or return to their pre-diagnosis lives. This means they face fear of disease progression rather than recurrence, physical side effects that may not get better with time, financial strain from the permanent inability to work, facing stigma, and challenges finding meaning while living with a terminal diagnosis. In contrast, a review by Mirosevic et al. found that among individuals who had completed curative treatment, the most prevalent challenges were related to fear of recurrence and a desire for information about actions they could take to improve their health [27]. Unique to those with lung cancer, participants within this study discussed stigma related to their diagnosis and its association with smoking. Across all stages of lung cancer, patients experience impressions from themselves and others that their cancer is self-inflicted, regardless of smoking status [28,29,30,31]. This has led to concerns that their access to care as well as funding for lung-cancer related research will be limited due to public belief that they should be blamed for their cancer diagnosis [30,31]. As a result, research has shown that lung cancer patients experience increased depression and distress related to perceived stigma, as well as reluctance and delays in help-seeking behaviors [28,29].

Other post-treatment concerns commonly highlighted in the literature include difficulties returning to work, loss of connections to oncology care teams, navigating personal relationships as a survivor, feelings of abandonment following treatment completion, and the process of adjusting to a “new normal” [32,33,34]. Given that the population of individuals living long-term with mNSCLC is relatively new, it is unsurprising that most existing supportive care interventions have been designed for those completing curative treatment. For example, common interventions include physical exercise programs [35,36,37], typically offered short term (i.e., a number of weeks or months), survivorship care plans and guidelines to assist in transitions after treatment completion [38,39,40], and fear of recurrence interventions [41,42]. Individuals with mNSCLC may not be eligible for or benefit from these programs as they face ongoing treatment and adaption to life with advanced cancer, rather than recovery and reintegration after treatment.

Individuals treated for lung cancer with targeted therapy or immunotherapy have described themselves as “living in the twilight zone”, feeling too sick to fit in with healthy individuals, yet not sick enough to relate to those dying from cancer [43]. As such, interventions open to all individuals (such as support groups) may actually increase distress as participants with metastatic cancer may feel isolated and avoid sharing their experiences for fear of scaring those with earlier-stage cancers [44]. In contrast, supports focused on metastatic cancer often focus on accepting and preparing for the end of life [45,46,47,48], which many individuals on targeted or immune therapy will not need for several years. This highlights another gap in care uniquely affecting those treated for mNSCLC, and the need for targeted interventions for this population.

Aside from simply developing these supports, participants in this study highlighted that supports must be accessible and offered as a part of a suite of services provided to mNSCLC patients after their diagnosis. This consideration is well-founded, given that barriers to accessing supports have been consistently documented across Canada [24,48,49,50]. Canadian cancer patients within our study and others report that they are unaware of the resources available to them, often having to do their own research to access supports without guidance from their HCPs [50]. This appears to be a systemic issue throughout North America. Supportive care is often poorly integrated with oncology making it difficult for HCPs to connect patients with these resources [51,52,53]. In addition, there are several barriers to implementing supportive care resources into practice long term including limited funding, inadequate staffing, and competing priorities [54,55,56]. As such, future interventions designed for this population must take into consideration local resources and constraints to develop a model that is sustainable and accessible long-term. Of note, both research funders and researchers in Canada have begun to address such implementation considerations in their intervention research, with implementation science an embedded part of funding programs and funded projects [57,58,59]. Without efforts to implement needed supports, the growing population of individuals living long-term with mNSCLC will continue to experience preventable challenges.

Given the lack of literature in this area, this study was exploratory in nature with an initial recruitment target of 8–10 participants. Our final sample size was 8, which is relatively low, even for qualitative research. Still, by the end of the eight interviews, the main challenges, barriers, and proposed changes discussed by participants were prevalent across the dataset. The dataset was rich, with participants providing detailed narratives and descriptions of their experiences living with mNSCLC. Although we cannot state with certainty that data saturation was achieved, others have found that saturation can be achieved with as little as nine to twelve participants [60,61], and that the basic features of meta themes are present with as few as six interviews [60]. Moreover, Sandelowski advises that the sample size in qualitative research should be large enough to capture a “new and richly textured understanding” of participant experiences yet small enough to permit the “deep, case-oriented analysis” representative of qualitative inquiry [62]. While individual experiences will undoubtedly vary, this study provides critical insight into the experiences of, and challenges faced by, people living long-term with mNSCLC as well as considerations related to developing (or adapting) interventions for this growing population of Canadians.

## 5. Conclusions

Individuals living long term with mNSCLC experience several challenges that are distinct from cancer patients treated with curative intent. Despite the significant impact these challenges have on their daily life, this population continues to face barriers to accessing resources to support them. To better support this growing population, oncology care must move beyond disease management to better integrate with supportive care services. Developing informational tools, health promotion programs, and navigational supports is critical to address these gaps and help individuals manage life on long-term cancer treatment.

## Figures and Tables

**Table 1:** Demographic Characteristics.

Demographic	*n* (%)
*Age (Years)*	
Mean (range)	59.5 (40–77)
*Years since diagnosis*	
Mean (range)	4 (1–11)
*Gender*	
Male	3 (37.5)
Female	5 (62.5)
*Residence type*	
Rural	2 (25.0)
Urban/Suburban	6 (75.0)
*Education level*	
College or Bachelor’s degree	4 (50.0)
Master’s degree or Doctorate	4 (50.0)
*Distance from cancer specialist*	
<30 min	1 (12.5)
30 min–1 h	5 (62.5)
>1 h	2 (25.0)

## Data Availability

The data presented in this study are available on request from the corresponding author. The data are not publicly available due to privacy and confidentiality considerations.

## Supplementary Materials

The following supporting information can be downloaded at: https://www.mdpi.com/article/10.3390/curroncol32100570/s1, File S1: Interview Guide.

## Abbreviations

The following abbreviations are used in this manuscript:

ALK+	Anaplastic Lymphoma Kinase
HCP	Healthcare Provider
mNSCLC	Metastatic Non-Small-Cell Lung Cancer
