# Supplementary material for: Exploring the Experiences of Individuals Diagnosed with Metastatic Non-Small-Cell Lung Cancer: A Qualitative Study
Source: Curr Oncol. 2025 Oct 15;32(10):570. doi: 10.3390/curroncol32100570 (PMC12564169; doi:10.3390/curroncol32100570)
Supplement: Supplementary file 1 [file curroncol-32-00570-s001.zip › curroncol-3899603-supplementary.pdf]

Supplementary File S1: Interview Guide

PREAMBLE [*explained to the participant by the interviewer following introductions]*

The purpose of the interview today is to learn about your experiences living with mNSCLC, any needs you have faced as a result of your cancer and/or treatment, as well as how these needs have been met (or not). We also want to ask you about your thoughts on key policies and features that need to be considered when providing supports for this population. Our goal is to inform future studies that will focus on developing interventions to meet the needs of those living with mNSCLC. Do you have any questions before we get started?

Background/Demographic information

- What is your age?
- What is your gender identity?
- Do you live in a rural or urban area? How far do you have to travel for medical care?
- What is the highest level of education you have obtained?
- When were you diagnosed with mNSCLC?
- What type of treatment/therapy did you receive/are you receiving?

Healthcare Experiences

- Tell me about your healthcare experiences since beginning treatment for mNSCLC
    - Which care providers do you see?
    - How frequently do you see them?

Needs resulting from mNSCLC

- Since beginning treatment for mNSCLC, what issues have you experienced?
    - Physical symptoms (i.e., pain, fatigue, nerve problems, etc.)
    - Emotional/psychosocial issues (i.e., anxiety/stress, depression/sadness, relationship issues with family/partner/friends, fear of cancer progression, uncertainty around prognosis, stigma associated with diagnosis, etc.)
    - Practical issues (i.e., loss of job, getting to and from appointments, costs associated with healthcare, taking care of family, etc.)

- Have these concerns/issues been addressed by your care providers?
  - If so, how?
  - If not, why?
  - Has anyone else provided supports for these needs?
- Have you accessed any supports outside of your regular care providers?
  - How did you find these supports? Did you hear about them from a clinician or did you seek them out on your own?
- Are there any specific needs that you have experienced that you have not been able to address?

Factors to consider in developing supportive care interventions

- If you could describe the best possible care that you could receive, outside of your cancer treatment specifically, what would that look like?
- Is there anything that has made it harder or easier to receive support for your needs?
  - What about factors related to your own personal situation that have impacted your ability to access support? (e.g.., travel, costs, pain, support from family, etc.)
- If programs or services were developed to meet the needs of those living with mNSCLC, which factors would need to be considered when designing them?
  - What would the ideal program look like to you?
  - What information about mNSCLC would you want to be provided to you?

Closing questions

- Is there anything else you want us to know about your experiences living with mNSCLC?
- Is there anything else that should be considered when designing supportive care programs and services for this population?
